# PARP Inhibitors and Radiometabolic Approaches in Metastatic Castration-Resistant Prostate Cancer: What’s Now, What’s New, and What’s Coming?

**DOI:** 10.3390/cancers14040907

**Published:** 2022-02-11

**Authors:** Andrea Marchetti, Matteo Rosellini, Giacomo Nuvola, Elisa Tassinari, Veronica Mollica, Alessandro Rizzo, Matteo Santoni, Alessia Cimadamore, Andrea Farolfi, Rodolfo Montironi, Stefano Fanti, Francesco Massari

**Affiliations:** 1 Medical Oncology, IRCCS Azienda Ospedaliero-Universitaria di Bologna, Via Albertoni 15, 40138 Bologna, Italy; andrea.marchetti12@studio.unibo.it (A.M.); matteo.rosellini@studio.unibo.it (M.R.); giacomo.nuvola@studio.unibo.it (G.N.); elisa.tassinari@studio.unibo.it (E.T.); francesco.massari@aosp.bo.it (F.M.); 2 Struttura Semplice Dipartimentale di Oncologia Medica per la Presa in Carico Globale del Paziente Oncologico “Don Tonino Bello”, IRCCS Istituto Tumori “Giovanni Paolo II”, Viale Orazio Flacco 65, 70124 Bari, Italy; a.rizzo@oncologico.bari.it; 3 Oncology Unit, Macerata Hospital, 62100 Macerata, Italy; mattymo@alice.it; 4 Section of Pathological Anatomy, School of Medicine, Polytechnic University of the Marche Region, United Hospitals, 60126 Ancona, Italy; a.cimadamore@staff.univpm.it; 5 Nuclear Medicine Unit, IRCCS Azienda Ospedaliero-Universitaria di Bologna, University of Bologna, 40138 Bologna, Italy; andrea.farolfi@aosp.bo.it (A.F.); stefano.fanti@aosp.bo.it (S.F.); 6 Molecular Medicine and Cell Therapy Foundation, Department of Clinical and Molecular Sciences, Polytechnic University of the Marche Region, 60126 Ancona, Italy; r.montironi@staff.univpm.it

**Keywords:** DDR, Lu-PSMA, mHSPC, mCRPC, PARP inhibitors, prostate cancer, radium-223, radiometabolic

## Abstract

**Simple Summary:**

Prostate cancer still represents an important health problem in men, considering its high frequency. Over the last decade, novel treatment options have emerged, leading to notable clinical benefits. These recent scientific acquisitions are creating the basis to widen the treatment scenario of this tumor, evolving from targeting the androgen receptor axis or the traditional chemotherapy approach.

**Abstract:**

In recent years, the advances in the knowledge on the molecular characteristics of prostate cancer is allowing to explore novel treatment scenarios. Furthermore, technological discoveries are widening diagnostic and treatment weapons at the clinician disposal. Among these, great relevance is being gained by PARP inhibitors and radiometabolic approaches. The result is that DNA repair genes need to be altered in a high percentage of patients with metastatic prostate cancer, making these patients optimal candidates for PARP inhibitors. These compounds have already been proved to be active in pretreated patients and are currently being investigated in other settings. Radiometabolic approaches combine specific prostate cancer cell ligands to radioactive particles, thus allowing to deliver cytotoxic radiations in cancer cells. Among these, radium-223 and lutetium-177 have shown promising activity in metastatic pretreated prostate cancer patients and further studies are ongoing to expand the applications of this therapeutic approach. In addition, nuclear medicine techniques also have an important diagnostic role in prostate cancer. Herein, we report the state of the art on the knowledge on PARP inhibitors and radiometabolic approaches in advanced prostate cancer and present ongoing clinical trials that will hopefully expand these two treatment fields.

## 1. Introduction

Excluding skin cancer, prostate cancer (PCa) is still the most common tumor diagnosed in men. Despite the good prognosis in terms of 5-year survival rate considering all stages, reaching up to 98%, prostate cancer can threaten long-term health and still remains the second-leading cause of cancer related death in men, after lung cancer [1]. When approaching Pca, it would be reductive to define it as a single entity; it should more properly be considered an heterogenous condition, which ranges from a relatively indolent to an aggressive disease. Focusing on metastatic Pca, androgen deprivation therapy (ADT) remains the cornerstone of hormone sensitive phase (hormone sensitive prostate cancer—HSPC), which usually lasts about 2 years, thus progressing to a castration-resistant disease state (castration resistant prostate cancer—CRPC) [2]. Several mechanisms have been called into account to explain resistance to therapies, such as androgen receptor (AR) gain-of-function mutations or splice variants (e.g., AR-V7), loss of tumor suppressor genes (e.g., p53, pTEN), and modifications of stromal components into the tumor microenvironment, promoting invasion, neoangiogenesis, and metastatisation process [3]. During the last few years, great efforts have been made to extend therapeutic options for metastatic CRPC (mCRPC), providing to clinicians the availability of different agents capable of prolonging patients outcomes. Since 2004, docetaxel plus prednisone remains a viable first option in symptomatic patients with aggressive disease [4]. Cabazitaxel, a tubulin-binding taxane not cross-resistant with the previous one, has been approved as a possible second line after progression to docetaxel [5]. Given the pivotal role of androgenic signaling even during advanced disease stages, novel androgen receptor signaling inhibitors (ARSI), abiraterone acetate, and enzalutamide, have been introduced into the therapeutic scenario, before or after docetaxel chemotherapy [6,7]. Excluding direct or indirect crosstalk with androgen receptor pathway, other options have been investigated in pretreated mCRPC patients.

Among these, radiometabolic approaches have been explored, starting with the introduction of radium-223, a calcium-mimetic alpha emitter with a short range, that selectively binds to areas of increased bone turnover, such as skeletal metastases, considering the enhanced osteotropism of prostate cancer. The high-energy alpha particles can cause several double-stranded DNA breaks, leading to localized cytotoxic effect in the target lesions, with relatively low toxic effects on the nearby bone marrow [8]. It is well established the role of radium-223 in symptomatic patients with bone metastases; while the best timing of its administration and the possibility of combining radium-223 with other therapeutic agents used in mCRPC is far less well known. In fact, on one hand, the phase III trial ALSYMPCA showed the efficacy of this targeted α-emitter in this setting; however the detrimental effect of the combination of radium-223 and abiraterone in terms of bone health is also reported. In particular, in the ERA 223 trial, this combination did not improve symptomatic skeletal event-free survival in mCRPC men with bone metastasis [9,10]. Regarding the interesting results of the recent phase II TheraP trial, that compared Cabazitaxel versus Lu-PSMA-617, a beta emitter that binds a prostate-specific membrane antigen (PSMA), several potential alternatives are yet to arise [11].

Another important therapeutic field being investigated in mCRPC is based on the percentage of patients, estimated to be about a 23%, with deleterious alterations in genes involved in homologous recombination repair (HRR), mainly *BRCA1*, *BRCA2,* and *ATM* [12]. These aberrations make PCa a perfect candidate to poly-adenosine-diphosphate-ribose polymerase (PARP) inhibitors, thanks to several mechanisms. Primarily, the “synthetic lethality” induced by exogen blockade of base excision repair (BER) machinery, used by tumor cells to escape the HRR defect; the PARPi binds and “traps” PARP-1 enzyme on the chromatin, creating a damage necessitating HRR for its removal, and the enhancement of non-homologous end joining, which may elicit a tumoricidal effect [13]. Referring to this biological rationale, several studies investigate olaparib, an orally bioavailable PARP inhibitor approved for advanced ovarian and breast cancer, highlighting its antitumor activity in mCRPC in patients with specific genomic damage [14,15]. These new discoveries underline the growing need to implement patients’ stratification to better tailor the treatment algorithm on the patient genomic characteristics.

We performed a review on the very novel therapeutic fields under development and investigation in mCRPC scenario, in addition to the more explored and largely used ARSI. With this regard, we collected recent evidence and studies on the two main novel therapeutic acquisition that are increasingly entering in the treatment weapons at the clinician disposal in the recent years: PARP inhibitors and radiometabolic approaches. We reported studies supporting the rationale and clinical trials to present the current knowledge on these two approaches.

## 2. DNA Damage Repair Genes and PARP Inhibitors in mCRPC

### 2.1. Role of DNA Damage Repair Genes in Prostate Cancer

Over the years, DNA damage repair (DDR) genes became a prolific subject of research in the metastatic castration-resistant disease and their alterations are more common than previously recognized. As revealed in a large trial from Stand Up to Cancer—Prostate Cancer Foundation (SU2C-PCF), somatic mutations are detected in about the 23% of mCRPC, while germline ones in about 8% [16]. Among DDR genes, mutation of *BRCA2* is the most common event reported and men with this germline alteration have a lifetime-risk of 30% to be affected by PCa [17,18]. In addition, other frequently altered DDR genes in prostate cancer are *BRCA1*, *ATM*, *CDK12*, *RAD51C,* and *FANCD2* [17]. According to the whole-exome sequencing of 444 metastatic prostate adenocarcinoma samples, the tumor-suppressor *BRCA2* function was mainly impaired by deep deletions and truncating mutations; to a lesser extent, by amplifications. Missense mutations of unknown significance, truncating mutations and deep deletions are all involved in *ATM* activity alteration. The altered *BRCA 1* function, latest in order of frequency, is commonly due to amplifications [19]. Whenever a damage occurs, several systems may be activated in order to restore the integrity of the DNA and to avoid the trigger of apoptosis’ pathway. If the insult involves a single strand of DNA, Mismatch Repair (MMR), Single-Strand Break Repair (SSBR), Nucleotide Excision Repair (NER) and BER can fix the damage using the complementary strand as a template. In particular, NER is activated by the formation of pyrimidine dimers and intra-strand crosslinks, that are bulky lesions provoked by UV rays, polyaromatic hydrocarbons (such as benzopyrenes), or platinum salts. PARP-1 and -2 are two enzymes that participate in the detection of single-strand breaks and in the recruitment of other proteins involved in the DNA repair mechanism. Moreover, these proteins help to regulate the transcription [20]. When PARP-1 and -2 are inhibited by specific drug (PARP inhibitors, PARPi), the insult may involve even the other undamaged strand. In a competent cell, HRR and NHEJ (non-homologous end joining) play a key role in repairing the double-strand breaks. *BRCA1* and *2*, *ATM*, *PALB2*, *RAD51,* and *CHEK2* are the main genes of HRR system. Other less mentioned genes are also worthy of note, considering their direct or indirect involvement in HRR machinery, such as *BRIP1, BARD1, RAD51B, RAD51C, RAD51B, FANCL, FANCE,* and *POLQ* [21]. On the contrary, if there is an alteration in HRR system, double-strand breaks cannot be repaired and so the effect produced by PARPi can be fatal for the cell [20,22]. In addition, in case of alteration of DDR genes, the adducts of DNA created by platinum salts will not be fixed by the cell, enhancing the cytotoxic action of these compounds [23]. Despite this evidence, platinum chemotherapy is still not a standard in the treatment of mCRPC, except in the transition to small-cell carcinoma or in neuroendocrine tumor [24,25].

**Figure 1:**
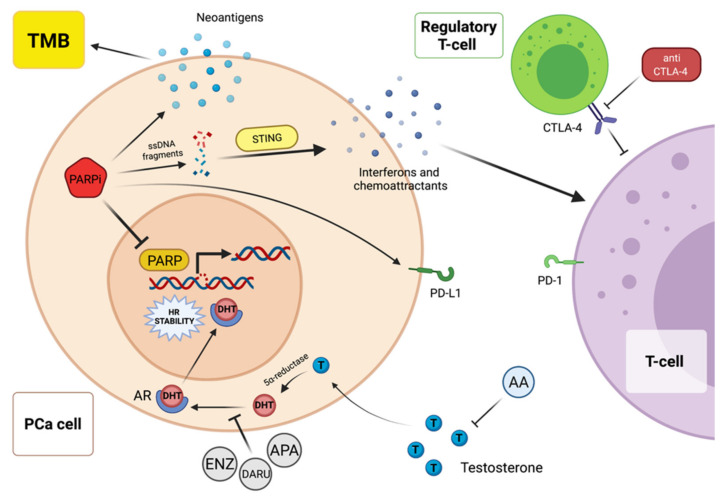
PARP inhibitors combined with immune checkpoint inhibitor are a promising treatment’ strategy on the basis of the close connection between these two pathways. PARP inhibition increases PD-L1 expression, neoantigens release and consequently tumor mutational burden. PARP inhibitors make tumor cells more sensitive to immunotherapy, promoting the release of ssDNA fragments that induce STING activation and the consequential liberation of interferons and chemo-attractants. In this way, the activation and the recruitment of T-cells is amplified. Similarly, the combination of PARP and ARSI is in the spotlight of researchers. The inhibition of the AR by novel anti-androgens alters the HRR stability, sensitizing the tumor cell to PARP inhibitors. Abbreviations: AR = androgen receptor, DHT = dihydrotestosterone, ENZ = enzalutamide, APA = apalutamide, DARU = darulotamide, TMB = Tumor Mutational Burden, ssDNA = single-strand DNA, STING = stimulator of interferon genes.

Prostate cancer cells can occasionally restore HRR in various ways. The most common event that induces PARPi resistance is the somatic mutation of a *BRCA1/2* allele, but also the methylation of the promoter of *BRCA1* and the reduction in expression of PARP-1 are other possible mechanisms [13,26]. Typically, *BRCA1* C61G mutation is less responsive to PARPi and it is common that these patients show an early resistance to this class of drugs [27]. With regard to the impact and prognostic role of DDR mutations, the prospective cohort study PROREPAIR-B was designed to explore this aspect. In this trial, 68 of the 419 eligible patients with mCRPC were characterized by germline mutations in DDR genes (the population was screened in 107 genes involved in the damage-repair process). Although this study did not meet the primary endpoint, which was the assessment of the impact of *ATM/BRCA1/BRCA2/PALB2* germline mutations on cause-specific survival (CSS) from the diagnosis of mCRPC, germline mutation in DDR genes were associated with faster switch to castration-resistant phase and with a trend to better response to ARSI and worse response to taxanes, particularly for *BRCA2* carriers [28]. PARPi are currently investigated in monotherapy and in combination with other compounds. Recently, several promising ongoing trials are testing PARPi combined with immunotherapy to enhance the response to immune checkpoint inhibitors (ICI). The rationale behind this approach derives from the ability of these compounds to increase programmed death-ligand 1 (PD-L1) expression, neoantigens release and, consequently, tumor mutational burden (TMB), and to release interferons and chemoattractants, which amplify T-cell’s activation and recruitment (Figure 1) [29,30]. A crosstalk between ARSI and PARPi is reported in the literature. As a matter of fact, AR inhibition compromises the HRR and may revert HRR status, resulting in an acquired sensibility to PARPi. In detail, a study published in 2013 on Cancer Discovery demonstrated that second-generation antiandrogen therapy downregulates the transcription of DNA repair genes, rendering the tumor responsive to PARPi, irrespective of HRR mutation status. (Figure 1) [20,31].

Moreover, PARPi impair the transcription mediated by AR and mutations in DDR genes are connected to the development and progression of PCa [32]. A study published in 2017 suggested that the potential use of PARPi combined with ADT could be effective before the development of CRPC, in advanced or high-risk patients [33].

### 2.2. PARP Inhibitors: What’s Now

Nowadays, PARPi are available weapons for the treatment of mCRPC with mutations in DDR genes, in particular in *BRCA1-2* and *ATM*. In the open-label, two-stage, single-group, phase II trial TOPARP-A, 50 patients received olaparib 400 mg twice a day. All were pretreated with docetaxel, 98% with abiraterone or enzalutamide, 58% with cabazitaxel, and 16 of 49 patients who could be evaluated had an alteration in DDR genes (only one man could not be assessed). Overall, 33% of the population and 14 patients of 16 who harbored DDR-deficiency had a response to olaparib. In addition, 14 of the 49 men showed a reduction to less than 5 cells/7.5 mL in the circulating tumor cell count and, among patients with measurable disease at baseline, 6 (19%) demonstrated a radiological partial response (PR) [34].

Successively, in another phase II study, TOPARP-B, 98 patients affected by mCRPC with alterations in DDR genes were randomized to olaparib at the dose of 300 or 400 mg BID. Confirmed response was achieved in 54% of patients who received 400 mg and in 39% of men who received 300 mg. Radiological response was reported in 24% of evaluable patients in the 400 mg group and in 16% of the evaluable ones in the 300 mg cohort. The reduction in PSA levels at least of 50% from baseline was achieved by 37% and 30% in the 400 mg and 300 mg arms, respectively, while circulating tumor cell count conversion was demonstrated in 53% and 48% of evaluable patients of the two corresponding groups. Patients with *BRCA1/2* alterations had a significant better radiological and PSA response if compared to patients who carried other DDR mutations (52% and 77%, respectively, for BRCA-mutated men versus 5% and 11% for patients who harbored other defects in DDR genes). About 37% of men in the 400 mg cohort developed toxicity that required a dose reduction to 300 mg, even though this group was characterized by higher benefit in terms of response [35]. PROfound is a phase III trial that enrolled 387 mCRPC patients pretreated with an ARSI (enzalutamide or abiraterone) and randomized to receive olaparib 300 mg or the other ARSI not prior employed [36]. Crossover to olaparib in case of progression was permitted. In total, 245 men were included in cohort A, characterized by *BRCA1/2* or *ATM* mutations, while cohort B consisted of 142 patients with alterations in other 12 genes involved in DDR, such as *RAD51B/C*, *CHEK1/2,* and *PALB2*. In cohort A, the primary endpoint imaging-based progression-free survival (PFS) was 7.4 months in the experimental arm versus 3.6 months in the control group (hazard ratio—HR—0.34, 95% confidence interval—CI—0.25–0.47, *p* < 0.001). In the overall population olaparib prolonged the imaging-based PFS (5.8 vs. 3.5 months, HR 0.49, 95% CI 0.38–0.63, *p* < 0.001). Men with *BRCA1* and especially *BRCA2* mutations had the biggest benefit from olaparib, as seen in an exploratory analysis. Although 2/3 of patients crossed over to olaparib after progression on ARSI, in the final analysis of overall survival (OS) the arm of cohort A treated with olaparib reached a median OS of 19.1 months versus a median OS of 14.7 months in the control group (HR 0.69, *p* = 0.02). For what concerns the median OS in the overall population, a significant benefit from olaparib was reported after the adjustment for crossover (17.3 months versus 14 months, HR 0.55, 95% CI 0.29–1.06) [14].

Another important study in this setting is TRITON2, a phase II, multicenter, open-label trial in which were enrolled 115 patients with mCRPC and *BRCA1/2* alteration (both measurable and not-measurable diseases were included) [37]. All had progressed after one or two lines of ARSI and one taxane. The aim of this trial was to evaluate the efficacy of rucaparib 600 mg BID in terms of objective response rate (ORR) and reduction in PSA at least of 50% from baseline. ORR per independent radiology review was 43.5% (95% CI, 31.0% to 56.7%; 27 of 62 patients) and per investigator assessment was 50.8% (95% CI, 38.1% to 63.4%; 33 of 65 patients). Notably, a comparable value of ORR was reported between men who harbored germline or somatic BRCA alterations and between patients with *BRCA1* or *BRCA2* mutations. PSA response rate was 54.8% (95% CI, 45.2% to 64.1%; 63 of 115 patients) and patients with *BRCA2* mutations presented a higher PSA response rate of compared to *BRCA1* carriers. In this trial were also enrolled 78 patients with deleterious non-BRCA DDR genes alteration. Alterations in *ATM*, *CDK12* and *CHEK2* were associated with limited radiological and PSA response to PARPi, while it was observed that alterations in other DDR genes (e.g., *PALB2*) may benefit from this class of compounds [38].

Niraparib at a dose of 300 mg daily was investigated in the single-arm, phase II GALAHAD trial in mCRPC men progressed to ARSI and a taxane-based chemotherapy. Patients were characterized by *BRCA1/2* alterations or mutation in *ATM*, *FANCA*, *PALB2*, *CHEK2*, *BRIP1,* or *HDAC2*. A pre-specified interim analysis revealed an ORR of 41% and radiographic PFS (rPFS) of 8.2 months in the population with *BRCA1/2*, while an ORR of 9% in patients non-BRCA1/2 mutated [39,40]. Moreover, another PARPi, talazoparib, was investigated in the single-arm, phase II trial TALAPRO-1. In this study, 127 patients with mCRPC and DDR genes alterations who had received at least a taxane in the metastatic setting and who had progressed to an ARSI, were treated with talazoparib 1 mg/die (in case of renal impairment 0.75 mg/die). After a median follow-up of 16.4 months ORR was 29.8% and anemia was the most common adverse event of grade 3/4 reported. On the basis of the result of this trial, other randomized phase III trials testing talazoparib are ongoing, as discussed later [41]. In Table 1, all the trials that assessed PARPi in mCRPC and discussed in this section are reported.

In conclusion, on the base of the results of PROfound trial and TRITON2, olaparib and rucaparib represent available therapies in mCRPC in selected patients with *BRCA1/2* or *ATM* alterations. Even though both studies highlighted the benefit of these compounds in this setting, several substantial differences in the study design can be pointed out. The most important ones are the differences in HRR mutations’ detecting methods and eligible genetic alterations, along with the divergence in the methods for assessing response and drug activity. In May 2020, olaparib received the approval by the Food and Drug Administration (FDA) for men with mCRPC progressed after abiraterone or enzalutamide and with germline or somatic mutation in *BRCA1, BRCA2, ATM, BARD1, BRIP1, CDK12, CHEK1, CHEK2, FANCL, PALB2, RAD51B, RAD51C, RAD51D,* and *RAD54L.* In September 2020, the European Medicines Agency (EMA) approved this compound in mCRPC patients who harbor *BRCA1/2* somatic or germline alterations, progressed after a prior ARSI. With regard to rucaparib, in May 2020, FDA approved this drug for mCRPC patients with a deleterious somatic or germline *BRCA* mutation who have been treated with androgen receptor-directed therapy and a taxane-based chemotherapy. Simultaneously, niraparib has recently received Breakthrough Therapy Designation by the FDA in order to accelerate the process of its approval.

## 3. Radiometabolic Approaches in mCRPC

### 3.1. Diagnostic Role

In recent years, the use of positron emission tomography (PET) in PCa is increasing. Several ligands are employed in clinical practice and others are being investigated, especially for the detection of early relapse after local therapy in a biochemical recurrence (BCR) setting, when PSA levels are rising. This is due to higher detection rate of local and distant relapse by PET imaging compared to standard imaging, such as a computed tomography (CT) scan and total bone scintigraphy [42,43]. The 18-fluorodeoxyglucose (FDG)-PET, differently to other types of cancer, has a limited role in PCa. Even if some studies suggest a possible value in detecting bone metastases or soft tissue metastasis in biochemical recurrence, its use is still controversial, especially with low level of PSA [44,45]. Conversely, 18-FDG-PET can be used to detect metastases and to monitor response to therapy in neuroendocrine prostate cancer [46].

The 18F fluciclovine-PET uses an ammino-acid analogue with increased uptake in cancer cells compared to normal tissue. In a retrospective analysis of 596 patients with suspected BCR, 18F fluciclovine-PET presented a detection rate of recurrent disease of 67.7%, with 38.7% in the prostate bed and 32.6% in the pelvic lymph nodes. Other metastatic sites were detected in 26.2% of patients [47]. A meta-analysis of 6 studies with 18F fluciclovine-PET showed an overall 87% pooled sensitivity and 66% pooled specificity in the detection of BCR [48]. However, the detection rate is lower compared to other tracers when PSA levels are lower than 2 ng/mL [43]. In the recently published phase II/III randomized EMPIRE-1 trial, 165 patients with detectable PSA after prostatectomy and negative conventional imaging were allocated to radiotherapy directed by conventional imaging alone or to conventional imaging plus 18F-fluciclovine-PET [49]. The primary endpoint of 3-year event-free survival (EFS) was 75% in the experimental arm versus 63% in the conventional imaging arm (difference 12.5; 95% CI 4.3–20.8; *p* = 0.0028).

The 11Carbon (11C) choline-PET is based on tracers targeting the lipid biosynthesis of the cell membrane, increased in cancer cells. A meta-analysis of 12 studies involving 11C choline-PET in BCR, showed a pooled sensitivity of 89% and pooled specificity of 89% [50]. Other two meta-analysis had similar pooled sensitivity and specificity, superior to 85%, in terms of both per lesion detection and per patient detection [51,52]. Both 18F fluciclovine-PET and 11C choline-PET are FDA approved for detecting relapse in BCR setting. Prostate-specific membrane antigen (PSMA)-PET uses radioligands directed to transmembrane PSMA protein, which is expressed 100 to 1000 times more in PCa than in normal prostate cells [53]. A systematic review and meta-analysis of 16 studies involving 68Gallium (68Ga) PSMA-PET showed an overall pooled per patient specificity and sensibility of 86% of this technique. Interestingly, a positive 68Ga PSMA-PET in patients with BCR was found also in a percentage of patients with low PSA level, with 42%, 58% and 76% positive rate in PSA levels of 0–0.2 ng/mL, 0.2–1 ng/mL and 1–2 ng/mL, respectively [54]. One prospective study compared PSMA-PET with other PET radiotracers in patients with BCR, showing higher detection rates with PSMA-PET, especially with low PSA levels (<2 ng/mL) [43,55].

18F-DCFPyL is another highly selective PSMA ligand. 18F-DCFPyL-PET has also been studied in BCR setting. In a prospective study, 130 patients with BCR had a positive detection rate from 60% with PSA level of ≥0.4 to <0.5 ng/mL, to 78% with a PSA level of ≥0.5 to <1.0 ng/mL, 72% with a PSA level of ≥1.0 to <2.0 ng/mL, and 92% with PSA level of ≥2.0 ng/mL [56]. The CONDOR trial is a phase III study that investigates the efficacy of 18F-DCFPyL-PET in BCR patients [57]. The trial reached the primary endpoint of correct localization rate (CLR), showing a CLR of 84.8%-87.0% and 63.9% of change in intended management due to PET results, further supporting the role of this radiotracer in men with recurrent disease. Both 68Ga-PSMA PET and 18F-DCFPyL PET are FDA approved for BCR setting after definitive local therapy. No definitive evidence is available on the role of the discussed PET-radiotracers in monitoring response to therapies for mCRPC or for baseline disease assessment in advanced stages and their application is under evaluation in clinical trials.

### 3.2. Therapeutic Role

Radium-223 dichloride (radium-223) is a targeting alpha emitter, that binds areas of high turnover in the bones, such as bone metastases. After the bond, it releases high energy alpha particles causing double-stranded DNA breaks as radiation induced damage. Its use in PCa is interesting, being an agent independent from the androgen-receptors pathway [58]. Phase I and II studies showed a good safety profile, with low rate of mielotoxicity. It also reduced bone pain, improved PSA and alkaline phosphatase (AP) trends, and appeared to improve survival in mCRPC [59,60]. A subsequent phase III ALSYMPCA trial randomized patients with mCRPC to receive radium-223 or placebo plus standard of care [61]. Patients enrolled had only bone metastasis (lymph nodes < 3 cm were permitted) and could have received or not docetaxel. The primary endpoint of OS favored the experimental arm resulting in 14.9 vs. 11.3 months of median OS compared to the control arm (HR 0.70; 95% CI 0.58–0.83) with 30% reduction in risk of death. All other secondary efficacy endpoint, such as time to skeletal related event, time to increased PSA, and AP, also favored radium-223. There was no statistical difference in hematological adverse events, such as myelosuppression. Given the positive results of the ALSYMPCA trial, radium-223 was also studied in combination with other agents. The ERA 223 trial investigated radium-223 in combination with abiraterone acetate in mCRPC. The primary endpoint was symptomatic skeletal event-free survival. Unfortunately, the combination not only did not improved efficacy, but also increased the risk of fracture [10]. Consequently, EMA restricted the use of radium-223 only in combination with LHRH analogues. Radium-223 was also studied in combination with docetaxel in patients with mCRPC and bone metastases in a phase I dose escalation/phase II randomized trial. The combination with the recommended phase II dose showed a good toxicity profile and improved the percentage of patients with PSA and bone formation biomarkers decline [62]. The DORA trial, a phase III trial of docetaxel in combination with radium 223 compared to docetaxel alone is currently ongoing (NCT03574571).

Lutetium-177 [177Lu]Lu-PSMA-617 is a radiolabeled molecule that binds to PSMA and releases high doses of β-particulate radiation, with high tumor specificity and limited damage to other tissues. [177Lu]Lu-PSMA-617 showed promising activity in patients with mCRPC progressed after standard therapies [63,64]. The LuPSMA trial is a single-arm phase II trial investigating the activity of 177Lu-PSMA-617 in patients with mCRPC progressed after standard treatments, such as docetaxel and second-generation anti-androgen agents. In total, 17 patients (57%) achieved a PSA decline ≥50% and 14 of 17 patients (82%) with CT evaluable target lesions achieved an objective response. Moreover, an improvement in pain and global health score was registered [65]. The TheraP trial is another phase II trial that randomized pretreated mCRPC patients to receive 177Lu-PSMA-617 or cabazitaxel. The primary endpoint of PSA reduction ≥ 50% was achieved in 66% versus 37% in the intention to treat population (*p* < 0.0001). The experimental arm also presented fewer grade 3–4 adverse events (33% vs. 53%) [11]. Recently, the results of the VISION study, the first phase III trial with 177Lu-PSMA-617, have been published [66]. Heavily pretreated mCRPC patients were randomized to receive 177Lu-PSMA-617 plus standard of care versus standard of care alone. The first of two alternate primary endpoints, image-based PFS, favored the experimental arm with 8.7 versus 3.4 months (HR 0.40; 99.2% CI, 0.29–0.57; *p* < 0.001). Additionally, OS was improved with the addition of 177Lu-PSMA-617 to standard of care, 15.3 versus 11.3 months (HR 0.62; 95% CI, 0.52 to 0.74; *p* < 0.001). Adverse events grade 3–4 were higher in the combination group 52.7% vs. 38% (Table 2).

## 4. Ongoing Clinical Trials: What’s Coming

### 4.1. PARP Inhibitors

Several ongoing trials are assessing the clinical efficacy and safety of PARPi alone or in combination with other agents, including ICIs and ARSI, in order to further expand the therapeutic landscape of metastatic prostate cancer toward a much more patient-based approach. It is known that, olaparib was the first PARPi evaluated in metastatic PCa patients [22]. With regard to the ongoing studies, the efficacy of olaparib alone or combined with abiraterone/prednisone versus abiraterone/prednisone is being tested in a phase II trial (NCT03012321) in chemotherapy-naïve mCRPC patients harboring loss of *BRCA1/2* or *ATM* genes on tumor biopsies. Of note, the administration of taxane chemotherapy in the hormone-sensitive setting is not an exclusion criterion if stopped at least 4 weeks prior to patient’s recruitment. Likewise, the phase III PROpel study (NCT03732820) is assessing the coadministration of abiraterone/prednisone with olaparib or placebo as frontline treatment for mCRPC. The primary end point is rPFS, while secondary endpoints are OS, time to first subsequent therapy or death, time to pain progression and health related quality of life (QoL). The presence of DDR genes’ mutations is not an including criterion for patients, but it is considered in the exploratory analyses [67]. The combination of a PARPi with an ARSI is a very promising strategy, in the light of the above-mentioned interplay between HRR system and hormonal treatments (Figure 1). In addition, recent data showed that enzalutamide or ADT could lead prostate malignant cells to a state of *BRCA*ness, with a resulting higher sensitivity to PARP inhibition [33,68]. On the basis of preclinical studies showing the synergistic effect of ICIs and PARPi [69], a phase I/II trial had already evaluated the association of olaparib and the anti-PD-L1 durvalumab in previously treated mCRPC patients [70]. Nowadays, the KEYNOTE-365 study (NCT02861573) is noteworthy. As a matter of fact, the cohort A of this phase Ib/II trial has the purpose to assess the clinical activity of olaparib combined with the anti-PD-1 pembrolizumab on 104 post-docetaxel mCRPC patients who progressed after at least two lines of ARSI (Table 3). Enrolled patients did not have detectable HRR gene alteration. Evan Yu and colleagues have recently displayed the interim results with a median follow up of 19.3 months of cohort A patients during the European Society for Medical Oncology (ESMO) Congress 2021. The antitumor activity of this combination was evaluated via PSA response rate (more than 50% from baseline value), which was 14.7% in cohort A patients. A disease control rate (DCR) of 26.5% and a confirmed ORR of 6.9% were also reported, unveiling a modest efficacy of olaparib plus pembrolizumab in molecularly unselected mCRPC patients [71]. Moreover, the prevalence of *BRCA* and HRR genes’ mutations in these patients has been highlighted in a concurrent biomarker analysis, as well as their association with the activity of this combination. Encouraging PSA response rates and ORRs were described in *BRCA*-mutated versus non *BRCA*-mutated patients (50% vs. 14% and 33% vs. 6%, respectively). Promising results were observed also in HRR-mutated patients [72]. Even though these data should be read with caution, due to the small sample size, the combination appeared to improve PSA response rate regardless of HRR status, if compared with olaparib or pembrolizumab monotherapies in the same setting [72]. The phase III KEYLYNK-010 trial (NCT03834519) will follow up on these results by comparing the combination of pembrolizumab and olaparib with a not previously received ARSI in mCRPC patients who have been treated with a taxane chemotherapy and abiraterone or enzalutamide. PFS and OS are the coprimary endpoints of this study [73].

As already pointed out, rucaparib gained FDA accelerated approval for mCRPC patients with *BRCA1/2* alterations who have previously received treatment with an ARSI and a taxane [74]. The subsequent full FDA approval will depend on TRITON-3 trial’s results. This ongoing randomized phase III study is comparing rucaparib with abiraterone/prednisone, enzalutamide, or docetaxel in mCRPC patients, harboring a deleterious germline or somatic *BRCA1*, *BRCA2*, or *ATM* mutation (NCT02975934). One prior ARSI-based therapy does not represent an exclusion criterion for enrolling patients, unlike the taxane chemotherapy. The primary endpoint is rPFS. The combination of rucaparib with the checkpoint inhibitor nivolumab has been tested in the phase II CheckMate 9KD trial (NCT03338790), and the related final analysis results have been newly presented at the ESMO Congress 2021. Enrolled patients were not selected based on HRR genes’ alterations, although homologous recombination deficiency (HRD) status was defined after recruitment [75]. Of note, HRD is a phenotypic behavior of malignant cells, which are characterized by alterations of the many proteins involved in the HRR system, and may be caused by germline and somatic *BRCA* mutations, as well as alterations of genes as *ATM*, *CHEK2*, *RAD51*, *MRE11A,* and so on, and epigenetic phenomena [76]. Adding nivolumab to rucaparib showed increased ORR and PSA response rate (respectively, 25% and 41.9% with a 17.5 months-long median follow up) among chemotherapy-naïve mCRPC men with HRR deficiency positive (HRD+) tumors. On the other hand, the clinical activity of this combination in HRR deficiency negative (HRD-) patients was limited. As for secondary endpoints, median OS and median PFS were higher among HRD+ than HRD- patients [75]. In CheckMate 9KD, the tissue-based evaluation of HRDstatus has been completed using the FoundationOne test (Foundation Medicine Inc, Cambridge, MA, USA).

In addition, the phase III CASPAR trial (NCT04455750) is comparing the combination of enzalutamide with rucaparib or placebo in 1002 untreated and molecularly unselected patients, with a planned completion by September 2026. Coprimary endpoints are rPFS and OS.

Among the remaining PARPi, niraparib is characterized by higher potency than other agents: it is a selective inhibitor of PARP-1 and PARP-2 enzymes, with a long half-life of 36 h, letting the once-daily administration. As already discussed, the use of niraparib as monotherapy is being evaluated in the ongoing phase II GALAHAD study (NCT02854436) with encouraging preliminary results [39]. More recently, niraparib was shown to ensure an improved and more durable health-related QoL, with a better pain control, especially in *BRCA*-mutated patients [77]. The phase III MAGNITUDE trial (NCT03748641) is currently recruiting mCRPC patients to compare the addition of niraparib or placebo to abiraterone/prednisone as first line treatment strategy. HRR mutational status will be used for randomization; the primary endpoint of the study is rPFS. The TALAPRO series of clinical trials is investigating the other PARP-1/2 selective inhibitor talazoparib to treat men with metastatic prostate cancer. The favorable results of the TALAPRO-1 trial [41] supported further and larger randomized clinical trials to define the role of talazoparib in mCRPC patients without detectable DDR gene mutations, too. The comparison between the combination of enzalutamide plus talazoparib and enzalutamide alone is under evaluation in the randomized phase III TALAPRO-2 study (NCT03395197), which is recruiting both molecularly selected and unselected mCRPC treatment-naïve men. Coprimary endpoints are rPFS along with the confirmation of the talazoparib dose (0.5 mg/die) based on its safety profile. The estimated completion date is November 2024.

**Figure 2:**
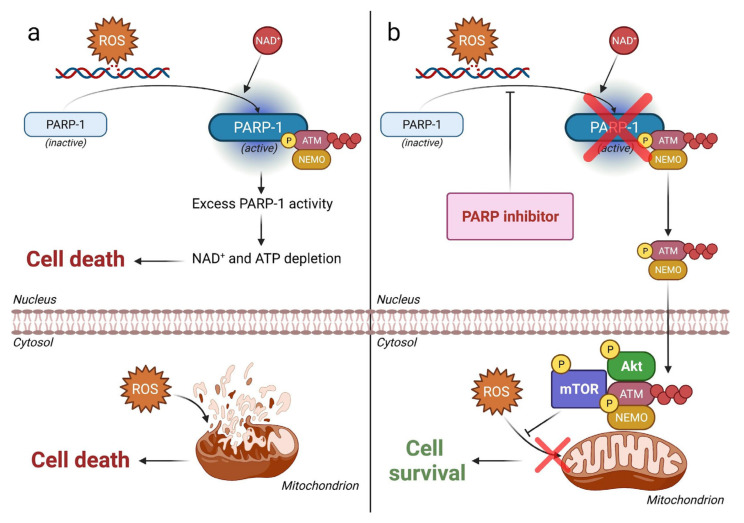
Among the many PARPi resistance mechanisms, the PARP-1/Akt interaction in oxidative stress could explain the potential efficacy of PARPi + AKT-inhibitor combinations. (**a**) Regularly, the nuclear PARP-1 enzyme is activated by oxidative stress causing DNA strand breaks. The excessive PARP-1 activity depletes the substrate NAD^+^, thus exhausting ATP stocks and restraining ATM-NEMO complexes in nucleoplasm. Consequently, the oxidative stress is able to injure mitochondria, without the ATM-NEMO induced activation of Akt, leading to cell apoptosis. (**b**) In the presence of PARP inhibition, nuclear NAD^+^ and ATP stocks’ depletion is avoided, due to the blocked PARP-1 activity. This PARP inhibition lets activated ATM-NEMO complex to translocate to the cytosol, assembling the ATM-NEMO-Akt-mTOR cytoprotective signalosome in the outer membrane of mitochondria. The mitochondrial oxidative damage may be prevented due to the above-mentioned mechanism, allowing cell survival. Therefore, the inhibition of the Akt pathway may prevent this kind of PARPi resistance [79]. In the figure: pointed arrows represent activation pathways; arrows with flat ends represent inhibition pathways. Abbreviations: ROS = reactive oxygen species; P = phosphorylated; NAD^+^ = nicotinamide adenine dinucleotide; ATP = adenosine triphosphate.

The addition of other agents than immunotherapies or next generation hormonal therapies to PARPi is being assessed in several trials, enrolling mostly molecularly unselected mCRPC patients. Notably, the NCT03840200 and NCT02893917 trials are investigating the coadministration of rucaparib and the AKT-inhibitor ipatasertib, as well as the combination of olaparib and the pan-vascular endothelial growth factor receptors (VEGF-Rs) tyrosine kinase inhibitor cediranib, respectively. In the near future, these studies will provide interesting data regarding these promising combinations, whose rationale is to enhance the antitumor activity of PARPis [78,79]. In more detail, the PI3k-Akt pathway inhibition could be an optimal approach to overwhelm the PARPi resistance issue (Figure 2). Further exploratory analyses could point out in future which biomarkers are potentially associated with a better response to these combination therapies, thus increasing our knowledge in terms of precision medicine for PCa patients. The combination of PARP inhibition and ATR inhibition is a new therapeutic strategy, which is today assessed in different forms of cancer. The TRAP trial (NCT03787680) is an ongoing phase II study comparing the responses of mCRPC patients with DDR mutations to those of mCRPC patients without DDR mutations, who are all treated with olaparib plus the ATR-inhibitor AZD6738 [80]. Combination trials with PARPi and radiometabolic agents are discussed below in the paper.

Lastly, a current challenge for physicians is to better understand the role of PARPi in other clinical settings than the mCRPC, such as the metastatic hormone-sensitive or the non-metastatic castration resistant disease. For example, the ongoing TRIUMPH study (NCT03413995) is nowadays enrolling metastatic HSPC patients with germline HRR genes’ mutations in order to define the clinical activity of rucaparib monotherapy as an alternative to frontline ADT or other hormonal therapies. Furthermore, the efficacy of the talazoparib and enzalutamide combination in HRR mutated metastatic HSPC men is under investigation in the phase III TALAPRO-3 study (NCT04821622), whose design has been recently presented by Agarwal and colleagues at the ESMO Congress 2021 [81]. In the next few years, the efficacy of PARPi as earlier-line treatment will be confirmed in high-risk non-metastatic/localized PCa patients (either as monotherapy in biomarker-selected patients or in combination with ADT and radiotherapy in molecularly unselected patients), on the basis of many awaited underway trials [74]. Other issues to further solve to completely define how PARPi can personalize and improve the actual standard of care of prostate cancer include: the better definition of these agents’ sensitivity in the context of specific mutations (*BRCA1/2*, *ATM*, non-canonical *DDR* genes), the relevance of germline versus somatic and monoallelic versus biallelic HRR genes’ mutations, understanding how the current combination strategies may help to overwhelm the PARP-inhibition resistance and the comparative efficacy and safety of available PARPi. The answer to all these questions will provide new opportunities for precision oncology in prostate cancer. A summary of the ongoing clinical trials assessing PARP-inhibitors as monotherapy or in combination with other treatment agents in prostate cancer is reported in Table 3.

### 4.2. Radiometabolic Treatments

During the ESMO 2021 Congress, Morris has presented the design of the ongoing phase III PSMAfore trial (NCT04689828), in which the efficacy in terms of rPFS with either 177Lu-PSMA-617 or a change in ARSI is investigated among taxane-naïve mCRPC patients, who have progressed on one prior ARSI. Eligible patients may have a confirmed PSMA expression by [68Ga] Ga-PSMA-11 PET/CT [82]. In the current days, radiometabolic treatments are being assessed in combination with other agents for the treatment of metastatic prostate cancer, paving the way for potential upcoming combination strategies. For example, it has been suggested that radiometabolic therapies could have a synergistic antitumoral activity if added to ADT and PARP-inhibition. As a matter of fact, AR axis was shown to be activated by radiation-induced DNA double-strand lesions in PCa malignant cells, thus leading to the upregulation of several DDR genes [83]. The androgen-deprivation strategy may induce the downregulation of these DDR genes, promoting an increased malignant cell death. Therefore, the activity of PARP is increased, and this latter phenomenon leads to the tumor-cell survival and the modulation of AR axis activity [33]. This is the biological rationale of the combining use of PARPi, ADT, and radiation-based therapies.

Two early phase clinical trials for molecularly unselected patients with mCRPC are currently testing the combination of PARPi with the radium-223 (Table 3). The safety profile of niraparib and radium-223 is the primary endpoint of the ongoing single-arm NiraRad study (NCT03076203), which recruited 14 patients with mCRPC and bone metastases. On the other hand, the phase I/II COMRADE trial (NCT03317392) is evaluating radium-223 with or without laparib in 112 patients with bone metastases. Both are expected to be completed by November 2021. Likewise, the phase I LuPARP trial (NCT03874884) is recruiting paucisymptomatic mCRPC men who were already treated with an ARSI and docetaxel in order to define the safety of laparib and ^177^Lu-PSMA-617 (Table 3). Estimated study completion date is in October 2022. In the last few years, a novel class of radiopharmaceuticals (the so-called “rad-hybrid PSMA ligands” or “rhPSMA”) has been developed by Wurzer and colleagues [84,85]. Due to their specific features, these rhPSMA ligands can be evaluated both for imaging and therapy in prostate cancer. As a matter of fact, this new type of theranostic PSMA-targeting agents allows fast radiolabeling with ^18^F and radiometals and can be used to bridge imaging and treatment. Among those studied so far, ^18^F-rhPSMA 7 offers high detection rates in early biochemical recurrence after radical prostatectomy, working as a PET/CT ligand, mainly in patients with low PSA levels [84]. To date, the two phase III SPOTLIGHT and LIGHTHOUSE trials (NCT04186845 and NCT04186819, respectively) are investigating the safety and diagnostic performance of rhPSMA 7.3 (^18^F) PET ligand in men with suspected recurrence or newly diagnosed prostate malignancy (Table 4).

## 5. Conclusions

Prostate cancer is not a single entity, and the presence of specific molecular alterations is opening novel treatment fields. These recent scientific acquisitions are creating the basis to widen the treatment scenario of this tumor, evolving from targeting the androgen receptor axis or the traditional chemotherapy approach.

PARP inhibitors are showing an impressive potential in treating mCRPC, and several ongoing trials will shed light on their application in other settings than the pretreated metastatic castration-resistant disease.

Moving forward, combining PARPis with other agents (such as ICIs, ARSIs, or VEGF-R TKIs) represents a promising strategy against prostate cancer. Many mechanisms of PARPi resistance have been studied so far and some of these combinations could help us to overcome them. Nevertheless, many open issues still remain, that will be central topics for future research. Pivotal importance has to be addressed to the individuation of predictive biomarkers of response and further efforts should be made to identify specific patient’s characteristics that will support the choice of one treatment over the other, especially in the presence of multiple therapeutic weapons with profoundly different mechanisms of action. Moreover, an improved knowledge of the HRD cancers’ biology will lead to further approaches to avoid or delay PARPi resistance, thus gaining better long-term outcomes for our patients. Lastly, the greatest efficacy of PARPis has been shown in mCRPC patients with HRR and *BRCA1/2* mutations, whereas treatment benefits derived from PARPi-based combinations could be reported regardless of HRR status. Future data are awaited to better define how HRR status may affect physicians’ PARPi choice, and the role of other specific gene mutations. Determining the most accurate testing method for identifying an HRR mutation is another issue to overwhelm soon.

Radiopharmaceutical approaches also seem to be promising therapy options in some patients with metastatic disease. Even though their use is still limited, in the next few years we should witness a progression towards a more frequent use of ^177^Lu-PSMA-617 than radium-223, given the available recent results of the related studies. Future efforts are required to better stratify mCRPC patients who may benefit the most from these radiopharmaceutical agents or the related combination approaches.

## Figures and Tables

**Table 1:** Pivotal phase II or III trials assessing PARP inhibitors as treatment strategy in prostate cancer. Abbreviations: PARPi = poly (ADP-ribose) polymerase inhibitor; HRR = homologous recombination repair; HRD = homologous repair deficiency; DDR = DNA-damage response; mCRPC = metastatic castration-resistant prostate cancer; ARSI = androgen receptor signaling inhibitor; ORR = objective response rate; rPFS = radiological progression-free survival; mo = months; PRR = PSA response rate; HR = hazard ratio; CI = confidence interval.

Study (Authors, Year)	PARPi Tested	Phase	Setting	HRR Status Required for Inclusion	Primary Endpoints	Results
TOPARP-A (Mateo J. et al., 2015) [34]	Olaparib	II	mCRPC after 1 or 2 taxane- based regimens	No	Composite response rate	- All pts: 33%; - HRD pts: 88%.
TOPARP-B (Mateo J. et al., 2018) [35]	Olaparib	II	mCRPC after 1 or 2 taxane- based regimens	Bi-allelic deleterio- us *HRR* alterations	Composite response rate	*BRCA1/2*: 83.3%; *PALB2*: 57.1%; *ATM*: 36.8%; *CDK12*: 25%; other: 20%
PROfound (de Bono J. et al., 2020) [36]	Olaparib (vs. ARSI)	III	mCRPC after at least 1 ARSI	Bi- or mono- allelic, soma- tic or germline, deleterious *HRR* alterations (Cohort A: *BRCA1/2* or *ATM* mutations; cohort B: other 12 *DDR* genes mutations)	rPFS	- Cohort A: 7.4 mo vs. 3.6 mo (HR 0.34, 95% CI: 0.25–0.47); - Cohort B: 5.8 mo vs. 3.5 mo (HR 0.49, 95% CI: 0.38–0.63).
TRITON-2 (Abida W. et al., 2020) [37]	Rucaparib	II	mCRCP after at least 1 taxane-based regimen and 1 ARSI	Bi- or mono- allelic, soma- tic or germline deleterious *HRR* alterations	ORR and PRR	- Somatic *BRCA1/2*: ORR 43.9%/PRR 50.7%; - Germline *BRCA1/2*: ORR 42.9%/PRR 61.4%; - *ATM*: ORR 10.5%/PRR 4.1%; - *CDK12:* ORR 0%/PRR 6.7%; - *CHEK12*: ORR 11.1%/PRR 16.7%.
GALAHAD (Smith M.R. et al., 2019) [39]	Niraparib	II	mCRCP after at least 1 taxane-based regimen and 1 ARSI	Bi-allelic *HRR* or germline pathogenic *BRCA1/2* alterations	ORR	- *BRCA*: 41%; - Non-*BRCA*: 9%.
TALAPRO-1 (de Bono J.S. et al., 2021) [41]	Talazoparib	II	mCRCP after at least 1 taxane-based regimen and 1 ARSI	Mono- or bi-allelic *HRR* alterations (*CDK12* excluded)	ORR	- *BRCA1/2*: 50%; - *ATM*: 7%; - Other *HRR* genes: 0%

**Table 2:** Pivotal phase II/III trials of radiometabolic ligands in prostate cancer. Abbreviations: mCRPC = metastatic castration-resistant prostate cancer, AAP= abiraterone acetate + prednisone, SRE = skeletal related events, ARPi = androgen receptor pathway inhibitor, CT = chemotherapy, iPFS = imagine-based progression free survival, SoC = standard of care.

Classes of Compounds Tested	Study (Authors, Year)	Design	Patients Enrolled	Setting	Agent	Primary Endpoint	Results
Radium-223	ALSYM-PCA (Parker et al. 2013) [61]	Phase III, randomized	921	mCRPC with bone metastases	Radium-223 vs. Placebo	OS	14.9 vs. 11.3 months; (HR, 0.70; 95% CI, 0.58 to 0.83; *p* < 0.001)
	ERA 223 (Smith et al. 2019) [10]	Phase III, randomized	806	untreated mCRPC with bone metastases	Radium-223 + AAP vs. AAP	*SRE*	22.3 vs. 26.0 months (HR 1.122; 95% CI, 0.917–1.374; *p* = 0.2636)
	(Morris et al. 2019) [62]	Phase I to II, randomized	53	mCRPC with bone metastases	Radium-223 + Docetaxel vs. Docetaxel	PSA reduction > 50%	61% vs. 54%
177Lu-PSMA	LuPSMA (Hofman et al. 2017) [65]	Phase II, single arm	30	mCRPC after prior CT and at least one ARPi	177Lu-PSMA	PSA reduction > 50%	57% (95% CI 37–75)
	TheraP (Hofman et al. 2021) [11]	Phase II, randomized	200	mCRPC after prior CT and at least one ARPi	177Lu-PSMA vs. Cabazitaxel	PSA reduction > 50%	66% vs. 37% by ITT; (95% CI 16–42; *p* < 0·0001)
	VISION (Sartor et al. 2021) [66]	Phase III, randomized	831	mCRCP after at least 1 taxane-based regimen and 1 ARSI	177Lu-PSMA + SoC vs. SoC	iPFS, OS	iPFS 8.7 vs. 3.4 months (HR, 0.40; 99.2% CI, 0.29–0.57; *p* < 0.001) OS 15.3 vs. 11.3 months; HR, 0.62; 95% CI, 0.52–0.74; *p* < 0.001)

**Table 3:** Ongoing clinical trials of PARP-inhibitors in prostate cancer. Abbreviations: PCa = prostate cancer, mCRPC = metastatic castration-resistant prostate cancer, mHSPC = metastatic hormone-sensitive prostate cancer, nmCRPC = non-metastatic castration-resistant prostate cancer, ICI = immune checkpoint inhibitor, VEGF = vascular endothelial growth factor, AAP = abiraterone acetate/prednisone, enza = enzalutamide, ARPi = androgen receptor pathway inhibitor, RP = radical prostatectomy, ADT = androgen-deprivation therapy, RT = radiotherapy, CBDCA = carboplatin, CT = chemotherapy, pts = patients, ORR = objective response rate, OS = overall survival, rPFS = radiological progression-free survival, DLT = dose-limiting toxicity, AEs = adverse events, CR = complete response, PR = partial response.

Classes of Compounds Tested	Study	Design	Estimated Enrollment	Setting	Agent(s)	Homologous Recombination Repair Mutations	Primary Endpoint(s)
PARPi single agent	PROfound, NCT02987543	Phase III, randomized	340	mCRPC after one prior ARPi	Olaparib vs. enza/AAP	Selected	rPFS
	TRITON-3, NCT02975934	Phase III, randomized	400	mCRPC after one prior ARPi	Rucaparib vs. enza/AAP/docetaxel	Selected	rPFS
	BrUOG-337, NCT03432897	Phase II, single arm	13	Localized or locally advanced PCa (neoadjuvant setting)	Olaparib 300 mg/die Q4W up to 3 cycles, then RP	Selected	PSA response rate
	NCT03047135	Phase II, single arm, open label	50	Biochemically recurrent nmCRPC (prior RP required)	Olaparib	Unselected	PSA response rate
	PLATI-PARP, NCT03442556	Phase II, single arm	20	mCRPC after prior CT and ARPi	Rucaparib maintenance after 4 cycles of CBDCA + docetaxel chemotherapy	Selected	rPFS
	TRIUMPH, NCT03413995	Phase II, single arm	30	mHSPC	Rucaparib (as an alternative to ADT)	Selected	PSA response rate
	ROAR, NCT03533946	Phase II, single arm, open label	32	Biochemically recurrent nmCRPC	Rucaparib	Selected	PSA response rate
	GALAHAD, NCT02854436	Phase II, single-arm open label	301	mCRPC after prior CT and ARPi	Niraparib	Selected	ORR
	NCT04030559	Phase II, single arm	30	High-risk localized PCa (neoadjuvant setting)	Niraparib up to 3 cycles, then RP	Selected	PSA response rate
	TALAPRO-1, NCT03148795	Phase II, non-randomized	100	mCRPC after prior taxane-based CT and at least one ARPi	Talazoparib	Selected	ORR
PARPi + anti-androgen therapies	TALAPRO-2, NCT03395197	Phase III, randomized	872	mCRPC treatment-naïve	Talazoparib + enza vs. placebo + enza	Selected	Safety, PFS
	TALAPRO-3, NCT04821622	Phase III, randomized	550	mHSPC	Talazoparib + enza vs. placebo + enza	Selected	rPFS
	NCT03012321	Phase II, randomized	70	mCRPC treatment-naïve	Olaparib vs. AAP vs. olaparib + AAP	Selected	PFS
	PROpel, NCT03732820	Phase III, randomized	720	mCRPC treatment-naïve	Olaparib + AAP vs. placebo + AAP	Unselected	rPFS
	CASPAR, NCT04455750	Phase III, randomized	1002	mCRPC treatment-naive	Rucaparib + enza vs. placebo + enza	Unselected	rPFS, OS
	MAGNITUDE, NCT03748641	Phase III, randomized	1000	mCRPC treatment-naïve	Niraparib + AAP vs. placebo + AAP	Selected	rPFS
	NCT04037254	Phase II, randomized	180	High risk localized or locally advanced PCa (no prior treatments)	Niraparib + RT + ADT vs. niraparib alone vs. RT + ADT	Unselected	Maintenance of disease-free state
	ASCLEPIuS, NCT04194554	Phase I/II, single arm, open label	100	High risk locally advanced PCa (cN+)	Niraparib + AAP + leuprolide + RT	Unselected	DLT, biochemical failure (% of pts)
PARP + ICI	KEYNOTE- 365, NCT02861573	Phase Ib/II, non-randomized	1000 (104 in cohort A)	mCRPC after docetaxel and one prior ARPi	Olaparib + pembrolizu-mab (cohort A)	Unselected	PSA response rate, ORR, safety
	KEYLYNK-010, NCT03834519	Phase III, randomized	780	mCRPC after docetaxel and one prior ARPi	Olaparib + pembrolizu-mab vs. enza/AAP	Unselected	OS, rPFS
	NCT03810105	Phase II, single arm	32	Biochemically recurrent nmCRPC	Olaparib + durvalumab	Selected	Number of pts with undetectable PSA
	CheckMate 9KD, NCT03338790	Phase II, non-randomized	330	mCRPC chemotherapy-naïve	Nivolumab + rucaparib/enza/docetaxel	Selected	ORR, PSA response rate
	QUEST, NCT03431350	Phase Ib/II, multi-arm, non-randomized	150	mCRPC after prior CT and ARPi (depending on cohorts)	Niraparib + AAP vs. niraparib + JNJ-63723283 (anti-PD1)	Both selected and unselected	ORR, incidence of AEs
PARPi + radionuclides	LuPARP, NCT03874884	Phase I, single arm	52	mCRPC after prior CT and ARPis	Olaparib + 177Lu-PSMA	Not available	DLT, recommended phase II dose
	COMRADE, NCT03317392	Phase I/II, randomized	112	mCRPC after prior CT and ARPis	Olaparib + Radium-223 vs. Radium-223	Not available	rPFS, maximum tolerated dose
	NiraRad, NCT03076203	Phase Ib, single-arm	14	mCRPC after at least one prior ARPi, with or without prior CT	Niraparib + Radium-223	Unselected	DLT
PARPi + other molecules	TRAP, NCT03787680	Phase II, non-randomized	47	mCRCP after prior ARPi	Olaparib + AZD6738 (ATR-inhibitor)	Selected	Rate of response (CR or PR), PSA response >50% decline
	NCT03840200	Phase Ib, non-randomized	51	mCRPC after one prior ARPi	Rucaparib + ipatasertib (AKT-inhibitor)	Unselected	DLT, PSA response rate
	NCT02893917	Phase II, randomized	90	mCRPC after at least one prior therapy	Olaparib + cediranib (VEGF-R TKI) vs. olaparib	Not available	rPFS

**Table 4:** Ongoing clinical trials of radiometabolic ligands in prostate cancer. Abbreviations: mCRPC = metastatic castration-resistant prostate cancer, ARPi = androgen receptor pathway inhibitor, CT = chemotherapy, OS = overall survival, rPFS = radiological progression-free survival, DLT = dose-limiting toxicity, PPV: positive predicting value.

Classes of Compounds Tested	Study	Design	Estimated Enrollment	Setting	Agent(s)	Homologous Recombination Repair Mutations	Primary Endpoint(s)
Radium-223	DORA, NCT03574571	Phase III, randomized	738	mCRPC after prior ARPi	Radium-223 + Docetaxel vs. Docetaxel	Not available	OS
	COMRADE, NCT03317392	Phase I-II, randomized	112	mCRPC after prior CT and ARPi	Radium-223 + Olaparib vs. Radium-223	Not available	rPFS, maximum tolerable dose
	NiraRad, NCT03076203	Phase Ib, single arm	14	m CRPC after at least one prior ARPi	Radium-223 + Niraparib	Unselected	DLT
177Lu- PSMA	PSMAfore, NCT04689828	Phase III, randomized	450	mCRP C after prior ARPi	177Lu- PSMA vs ARPi	Not available	rPFS
	LuPARP, NCT03874 884	Phase I, single arm	52	mCRPC after prior CT and ARPis	Olaparib +177Lu- PSMA	Not available	DLT, recommended phase II dose
rhPSMA	SPOTLIGHT, NCT04186845	Phase III, single arm	319	Biochemical relapse	rhPSMA −7.3 (18F)	Not available	PPV
	LIGHTHOUSE, NCT04186819	Phase III, single arm	375	Newly diagnosed	rhPSMA −7.3 (18F)	Not available	Sensivity
